# Perspectives on the Management of Surplus Dairy Calves in the United States and Canada

**DOI:** 10.3389/fvets.2021.661453

**Published:** 2021-04-13

**Authors:** Katherine Creutzinger, Jessica Pempek, Gregory Habing, Kathryn Proudfoot, Samantha Locke, Devon Wilson, David Renaud

**Affiliations:** ^1^Department of Population Medicine, University of Guelph, Guelph, ON, Canada; ^2^Department of Animal Sciences, College of Food, Agricultural, and Environmental Sciences, The Ohio State University, Columbus, OH, United States; ^3^Department of Veterinary Preventive Medicine, College of Veterinary Medicine, The Ohio State University, Columbus, OH, United States; ^4^Atlantic Veterinary College, University of Prince Edward Island, Charlottetown, PEI, Canada

**Keywords:** dairy bull calves, animal welfare, veal, sustainability, calf health

## Abstract

The care of surplus dairy calves is a significant issue for the United States and Canadian dairy industries. Surplus dairy calves commonly experience poor welfare as evidenced by high levels of mortality and morbidity, and negative affective states resulting from limited opportunities to express natural behaviors. Many of these challenges are a result of a disaggregated production system, beginning with calf management at the dairy farm of origin and ending at a calf-raising facility, with some calves experiencing long-distance transportation and commingling at auction markets or assembly yards in the interim. Thus, the objectives of this narrative review are to highlight specific challenges associated with raising surplus dairy calves in the U.S. and Canada, how these challenges originate and could be addressed, and discuss future directions that may start with refinements of the current system, but ultimately require a system change. The first critical area to address is the management of surplus dairy calves on the dairy farm of origin. Good neonatal calf care reduces the risk of disease and mortality, however, many dairy farms in Canada and the U.S. do not provide sufficient colostrum or nutrition to surplus calves. Transportation and marketing are also major issues. Calves can be transported more than 24 consecutive hours, and most calves are sold through auction markets or assembly yards which increases disease exposure. Management of calves at calf-raisers is another area of concern. Calves are generally housed individually and fed at low planes of nutrition, resulting in poor affective states and high rates of morbidity and mortality. Strategies to manage high-risk calves identified at arrival could be implemented to reduce disease burden, however, increasing the plane of nutrition and improving housing systems will likely have a more significant impact on health and welfare. However, we argue the current system is not sustainable and new solutions for surplus calves should be considered. A coordinated and holistic approach including substantial change on source dairy farms and multiple areas within the system used to market and raise surplus dairy calves, can lead to more sustainable veal and beef production with improved calf outcomes.

## Introduction

Each year a portion of calves born on dairy farms are either unsuitable or not required to replace the milking herd, and these calves are commonly referred to as “surplus” animals. Surplus calves are 95% male (1–3) and the sale of these animals generally provides only a small percentage of income for dairy producers. The remainder of surplus calves are comprised of females not retained in the herd because enough replacement animals can be produced from 60% of the lactating herd (4) and infertile females that are a twin to a male calf. Historically, surplus dairy calves have been viewed as a “low-value by-product of the dairy industry” (5), which potentially results in surplus calves receiving poorer care than is given to calves perceived as “valuable” by dairy producers and calf raisers. In the United States and Canada, surplus calves are generally sold from the dairy farm of origin within days after birth, and common market destinations include “bob” veal (marketed <3 weeks of age and 150 lb), “formula-fed” or “special-fed” veal which accounts for the largest proportion of surplus calves (marketed at ~20 weeks of age) (6), or dairy-beef (marketed at 12–14 months of age) (7). Most surplus calves in the U.S. and Canada are raised for meat; however, it is not uncommon for calves to be euthanized on the dairy farm shortly after birth. Approximately 5% of dairy farmers in Canada reported euthanizing at least one male calf at birth (8). The driving factors of euthanizing male calves after birth is unclear; however, it is likely driven by the lack of market demand.

Historically, surplus calf production systems, including veal farms, have struggled with negative stigma, including societal concerns about animal welfare (9). Calves are removed from dairy farms as neonates (10), and are often transported long-distances with one or more stops at auction markets or assembly stations (11, 12) before arriving to calf raisers where they are housed individually and fed low planes of nutrition (13). Poor care surplus calves receive during the first few weeks of early life contributes to high rates of morbidity and mortality. Previous research has shown surplus dairy calves arrive to calf rearing facilities in the U.S. (11) and Canada (2) in poor health with signs of discomfort due to disease (14). Despite public scrutiny, little research has been done to determine best practices to promote the care of these animals. The research to date has mainly focused on characterizing problems within the current system, while less work has focused on corresponding solutions. The goals of this narrative review are to: (1) summarize current early life challenges of surplus dairy calves in the U.S. and Canada, (2) identify how such challenges originate and could be addressed, and (3) propose short- and long-term considerations for addressing these problems, including the development of an industry vision for how to manage these animals using perspectives from multiple stakeholders.

## Challenges With the Current Surplus Calf Production Chain

Surplus dairy calves face significant health and welfare challenges shortly after birth. Male surplus calves may be especially vulnerable to poor outcomes due to a lower standard of care after birth compared to female calves that remain in the herd as replacement animals (10, 15). Despite this, there is no mechanism for recording important aspects of calf care that may not be evident when they are marketed (e.g., colostrum provision and navel antisepsis). After leaving the dairy farm, calves often have long transport times and irregular feeding schedules (16). Additionally, commingling with unfamiliar animals from multiple sources and exposure to livestock markets are significant risk factors for disease spread (17). Thus, the early management of surplus dairy calves presents a significant risk to calf health, and such management practices are inextricably linked to their welfare and ability to thrive within veal and/or dairy-beef production systems.

### Management on Dairy Farms

Successful health outcomes for calves entering veal and dairy-beef production rely on appropriate husbandry on the dairy farm of origin. Calf care requires substantial time on dairy farms; however, it results in little to no immediate financial payoff, which is especially true for surplus dairy calves. For example, Wilson et al. (18) found for Canadian producers the necessary time and effort to care for newborn calves were barriers to the adoption of better management practices, especially for male calves. In interviews, dairy producers discussed an ethical desire to take good care of neonatal calves, but their actions were frequently misaligned (18). Specifically, male calves often receive worse care after birth than female calves on dairy farms. For example, Canadian studies have found males are more likely to receive colostrum with bacterial contamination (19) and lower volumes of colostrum (20) than female calves. Less research has been done in the U.S., however, (10) found male calves were more likely to be fed *via* different routes (e.g., suckling the dam) with a longer delay to the first feeding of colostrum. As a result of differences in colostrum feeding practices, male calves on Canadian dairy farms had lower serum total protein (used to determine successful passive transfer of immunity) than females (20). These results indicate improved colostrum feeding practices are needed for male calves.

In addition to FTPI, many calves experience health challenges. For example, 37% of male calves had at least one health abnormality such as diarrhea or navel disease at dairy farms before transportation to a calf raiser (21). This study further identified the importance of calf health on the dairy farm, as the presence of health abnormalities on dairy farms was significantly associated with subsequent mortality risk at the calf raiser.

### Transportation

Many countries have transportation regulations to meet the needs of young animals which may differ from mature animals. For example, (22) and the European Union (23) require that transported calves' navels are healed and dry and are a minimum age of 4 and 10 d, respectively. Comparatively, the U.S. and Canada have fewer requirements for animals in transport. Canada recently introduced new regulations that limits transport duration of pre-weaned calves to 12 consecutive hours before requiring access to food, rest, and water (24). The new Canadian regulations also state that calves shipped to assembly yards or auctions must be 9 days of age or older, and that calves have healed navels in order to be transported. In the U.S. pre-weaned calves fall under the transportation regulations established for all food production animals which limits transport to no more than 28 continuous hours (25).

There is a paucity of information regarding the distance or length of time calves are transported in the U.S. and Canada. A study in the U.S. estimated calves 7–10 days of age were transported between 450 and 977 km from livestock auctions in the northeast to calf raisers in Ohio (11). In the northwestern U.S. and western Canada, calves (animals weighing <275 kg) can be transported over 1,300 km (16). While exact transport durations have not been recorded, an expert panel reported that across Canada calves are frequently transported between 12 and 16 h (12). Outside of these studies, there is no research in the U.S. and Canada to our knowledge that describes the distance surplus calves are transported from dairy farms to livestock auctions and/or calf raisers.

Transportation includes multiple stressors such as handling, commingling with unfamiliar animals, exposure to new environments, food and water deprivation, and fluctuating temperatures (26). Feed and water deprivation during transportation likely contributes to the large number of calves that are dehydrated (27, 28) and in poor body condition (28, 29) upon arrival to calf raisers. Beyond physical changes, feed deprivation for long periods of time likely results in severe hunger and thirst. Expanding views of animal welfare include affective states, and to address these concerns, it may be prudent to better understand transport from the calf's perspective. Young calves may experience fear in response to novel environments (i.e., the trailer, auction, and new housing) and handling. An understanding of the calves affective state in response to transport should be used in conjunction with physical changes to inform future policy changes.

### Livestock Auctions

Livestock auctions are the most common destination for calves after leaving the farm. Roughly 40% of male calves born in the U.S. are sold through auctions, with the remainder sold directly to a calf raiser (30%) or dealer (18%) (30). Most small (68%) and medium (58%) sized herds sell surplus calves through an auction, whereas large farms more commonly sell calves directly to a calf raiser or another type of grower (30). Similar to the U.S., the majority of surplus calves in Canada are sold through auctions with a smaller proportion of calves sold directly to calf raisers (12). Marketing calves through auctions or other avenues was found to be largely dependent on region. Although auctions provide an avenue for buyers to visually assess animals, there are several significant health and welfare challenges that occur due to this method of marketing.

Livestock auctions represent a high biosecurity and infectious disease risk (31). Auction markets frequently assemble multiple livestock species, including adult cull cattle and neonatal calves, from different source farms in a common environment. Furthermore, most auction facilities cannot be effectively cleaned and disinfected, and thus, are a common point of direct or indirect transmission of infectious diseases. Multidrug resistant strains of *Salmonella* spp., a bacterium known for intestinal outbreaks in calf populations, including Dublin, Typhimurium, and Newport are common causes of disease outbreaks at veal and dairy-beef facilities (32). Surplus calves, which frequently have FTPI (11), are particularly at risk for infection. The exposure and infection of surplus calves at auctions facilitates the dissemination of pathogens that are important causes of disease in cattle and humans.

The health status of surplus calves delivered to livestock auctions likely influences both the spread of pathogens and subsequent disease susceptibility. At livestock auctions in Quebec and British Columbia, 43 and 21% of calves, respectively, had at least one health abnormality identified during a clinical exam (28, 33) which highlights that many calves arrive to auctions with health challenges. Health abnormalities included omphalitis, nasal or ocular discharge, depressed attitude, coughing, joint inflammation, and diarrhea. Omphalitis (characterized by navel swelling, discharge or evidence of pain) accounted for the greatest percentage of health abnormalities in both studies. Surprisingly, Marquou et al. (33) reported 12% of calves had neonatal characteristics (wet or difficulty standing) and 7% had wet umbilical stalks or navels, which may suggest calves arrive to auctions younger than previously reported. It is unclear if health abnormalities observed at livestock auctions begin during transport or on the dairy farm. Further research could help identify if transportation to auction markets contributes to development of health abnormalities observed in surplus dairy calves. Furthermore, livestock auctions negatively impact the affective state of young calves. Livestock auctions may not be equipped to routinely provide feed or water, and the abrupt commingling with unfamiliar animals causes additional stress. Wilson et al. (28) described the condition of pre-weaned calves at a livestock auction in British Columbia. The authors found calves did not have access to forage, milk, or water at the auction facility. Calves were housed in group holding pens, until the time of sale, then calves were most commonly placed in a sale ring alone. Once sold, calves were moved to a pen with access to a chute system that included a ramp to load calves onto a livestock trailer. Depending on the length of subsequent transport, calves likely experience long durations without access to milk or water; resulting in hunger and dehydration (34–36). Further, commingling with unfamiliar animals (37) and novel environments (38) are substantial social stressors for dairy calves.

### Calf Raisers

Surplus calves arrive to calf raisers in variable health condition, sometimes already experiencing respiratory or enteric disease (3, 11). Calves that arrive to veal facilities with health abnormalities are at greater risk of morbidity and mortality (2, 27), making calf health upon arrival important from both a welfare and productivity standpoint. Various strategies to combat disease have been utilized in the veal industry, including “all-in all-out” animal movement, individual housing, and prophylactic use of antimicrobials; however, our understanding of how such strategies can improve calf health needs to be further refined. Current industry practices and gaps in evidence-based best management practices are discussed throughout this section.

#### Calf Health

At the time of arrival to calf raisers, some calves suffer from poor body condition, navel inflammation, respiratory disease, or FTPI (11, 29, 39). Specifically, between 6 and 43% of male calves have FTPI upon arrival to calf raisers in the U.S. and Canada (11, 29, 39) and 30–60% of calves are clinically dehydrated (11, 39). Calves also frequently arrive to calf raisers in emaciated body condition or low body weight (29, 39). The presence of these health abnormalities, specifically low body weight, dehydration, and navel inflammation, are associated with increased risk of morbidity and mortality (1, 2, 27). Long durations without access to milk or water during transportation and at auction facilities, commingling, and variable care on the dairy farm of origin likely contribute to high rates of health abnormalities upon arrival to calf raisers. Importation of calves from multiple farms and commingling within livestock auctions also result in high infectious disease pressure. Severe outbreaks of clinical salmonellosis are relatively common, and result in high levels of mortality in calves (32). “All-in all-out” practices (raising calves in similarly aged groups) and other internal biosecurity measures are commonly utilized; however, the introduction of pathogens will be difficult to control as long as calves are routinely aggregated in livestock auctions prior to arrival at the calf-raiser.

**Figure 1:**
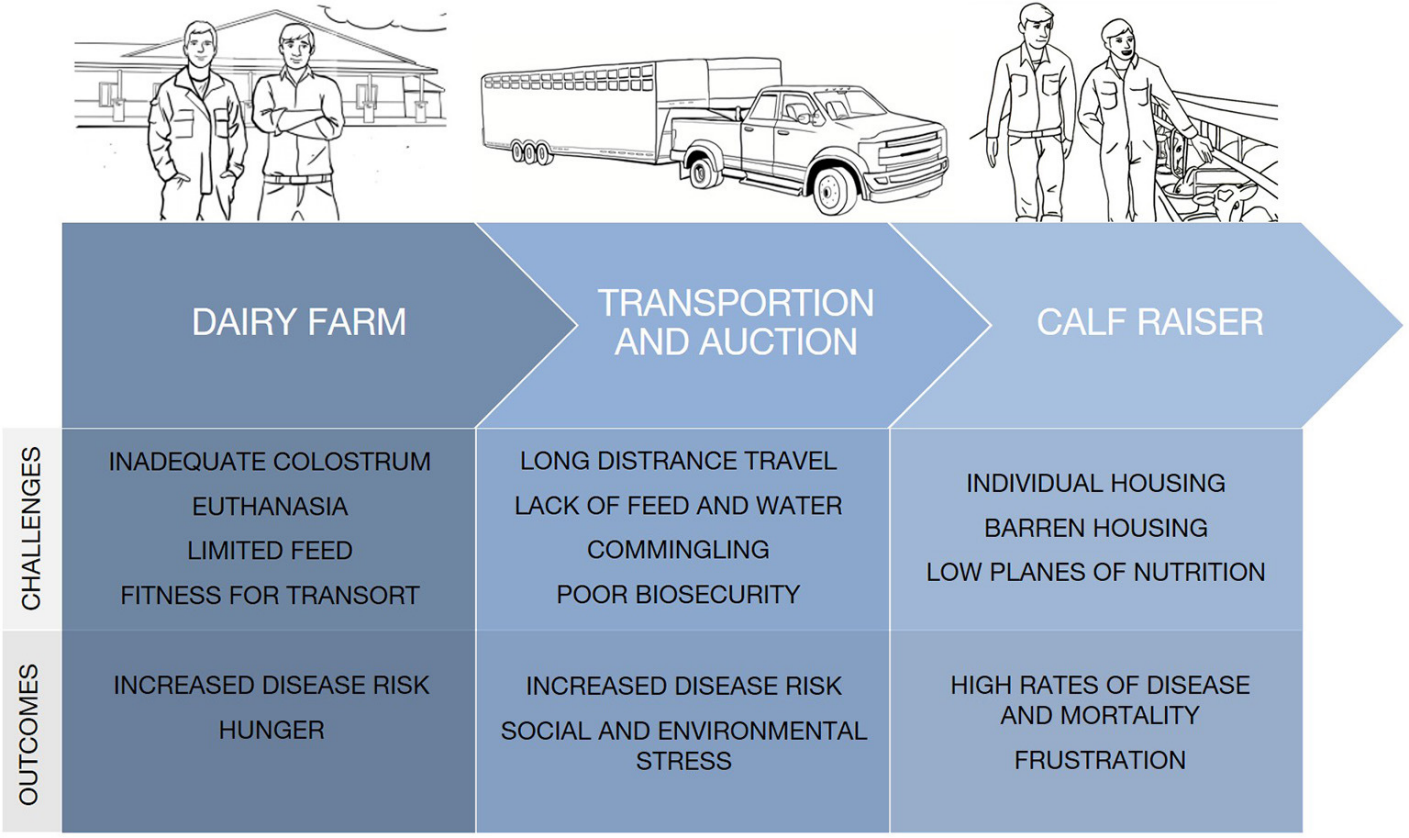
Challenges surplus calves experience from birth through early life management. Surplus calves frequently receive poor care on dairy farms of origin, then many calves are transported long distances and may be marketed through livestock auctions before arriving at the calf raiser. Each stage of the surplus calf production system presents unique challenges to calf health, affective states, and the ability to perform natural behaviors.

Given the multiple challenges from birth to arrival at calf-raisers (Figure 1), the first few weeks at the calf-raiser are a high-risk period. Over an 11 week period at a calf raiser in Ontario, 7.5% of calves died and almost 90% were treated with antimicrobials at least once for disease (27, 40). Scott et al. (27) and Renaud et al. (2) also found 68% of calves were treated with antibiotics and 42% of calf deaths, respectively, occurred within the first 3 weeks after arrival to calf raisers. Consistent with the previous studies, Winder et al. (1) found 7.6% of calves died over a 20 week period and the most common reasons for death included emaciation (21%), respiratory disease (16%), gastrointestinal causes (14%), and sudden death (13%). It is likely that the condition of calves on arrival is responsible for the high rates of early morbidity and mortality, however, death caused by emaciation and respiratory disease suggest that nutrition and housing strategies are inadequate. These causes of death also imply that there is a degree of calf suffering prior to death that needs to be addressed.

#### Antimicrobial Usage

Current antimicrobials use rates are influenced by high disease susceptibility and prevalent health abnormalities. For example, almost 90% of calves raised at a grain-fed veal facility in Ontario were treated with antimicrobials at least once during an 11-week period (27, 40). High rates of antimicrobial use has been associated with the development of antimicrobial resistance in commensal and pathogenic bacteria within the digestive and respiratory tract of veal calves (41, 42) and increased carriage of antimicrobial resistant bacteria in calf caretakers (43). Retail grain-fed and milk-fed veal products have also harbored antimicrobial resistant bacteria, highlighting the potential for negative public health consequences (44, 45). Similarly, antimicrobial use may be associated with the emergence of a multidrug resistant strain of *Salmonella* Heidelberg in dairy calves. The strain caused severe outbreaks in calf populations and a multi-state outbreak of salmonellosis in people that resulted in 56 illnesses and 17 hospitalizations (46). A direct effort to improve calf health should be made to reduce antimicrobial use, thus limiting the development of antimicrobial and multi-drug resistant pathogens.

#### Housing

Housing at calf raising facilities, particularly within the veal industry, has been criticized by the public [e.g., (47)] and animal welfare groups. It is commonplace to house calves individually with limited space for the first 8 weeks following arrival to calf raisers. Individual housing of calves is used a biosecurity measure to prevent respiratory disease [reviewed by (48)], which is a leading cause of morbidity and mortality in veal calves (49). However, the potential health benefits of individual housing are inconclusive as prolonged individual housing in veal facilities (>4 weeks) is a risk factor for nasal discharge and coughing (50). Furthermore, individual housing profoundly limits calves' ability to perform natural behaviors, such as play or social grooming (51, 52). Overall, housing calves in social isolation negatively impacts their physiology, behavior, and welfare [reviewed by (48)] likely due to the lack of both physical and social stimulation. A lack of stimulation may result in boredom and lead to the development of abnormal behaviors (53). In addition to socially restricted housing environment, access to the outdoors or pasture, under most circumstances, is not provided. Limited work has evaluated indoor vs. outdoor rearing systems. A recent study in Switzerland trialed the concept of an “outdoor veal calf” raising system and found a reduction in antimicrobial use and mortality (54). In this study, calves were not moved from the source dairy until 3 weeks of age and considerable effort was made to avoid livestock markets when sourcing calves, which mitigated major challenges faced by surplus dairy calves in the U.S. and Canada.

In some parts of the U.S. and Canada, public scrutiny has resulted in regulation and policies that impact how calves are housed and raised. For example, group housing after weaning and increased space allowance per calf is required in Canada (55) and by the Veal Quality Assurance program (56) in the U.S., as well as specific state legislation. For example, veal calves raised in California must have enough room to stand up, lie down, fully extend their limbs, and turn around freely (47). In addition, management practices that physically restrict animal movement, such as tethering, are prohibited through industry and legislative initiatives in the U.S. and Canada. Changes is housing systems are likely needed to reduce disease and promote the performance of natural behaviors. However, existing facilities may require significant adjustments to meet the needs of calves as some facilities are converted structures from old barns that were not designed to promote calf health and welfare (57).

#### Nutrition and Feeding

Feed programs must be designed to fit the nutritional needs of calves and delivered in a way that allows them to express natural behaviors. The volume of milk fed to surplus calves at calf raising facilities in the U.S. and Canada is unclear, however, it is likely low based on estimations from publications carried out on commercial veal facilities (58). In a 2010 survey of heifer raisers in the U.S., it was found that 76% of farms fed 1.89 L of milk twice daily per calf (59). Clearly, traditional limit feeding has many negative impacts on the calf. Specifically, when compared to traditional planes of nutrition (4 L of milk or less per day), higher planes of nutrition have been associated with improved immune function, resolution of diarrhea, and greater body weight gain (60–63). In addition to poor health and growth, limit feeding results in calf hunger. While no research has been performed on surplus calves, pre-weaned female calves fed <8 L of milk per day exhibit behavioral signs of hunger (64). An additional concern with certain special-fed veal calves, specifically for milk-fed veal calves, is the contribution of feeding strategies to abomasal damage, which has a prevalence at harvest ranging from 70 to 100% (65, 66). Abomasal damage is multifactorial in origin and could be due to inaccessibility to the outdoors and water, limited forms of roughage, bucket feeding, large and infrequent milk meals, and limited space allowance [reviewed by (13)]. Feeding calves low volumes of milk reduces productivity and health and leads to negative affective states, and it is unclear why the practice persists.

Along with providing low milk volumes in surplus calf production systems, milk delivery methods typically prohibit nutritive sucking behavior. Researchers have extensively documented the behavioral and physiological importance of sucking behavior for young calves (67, 68). Sucking deprivation results in frustration (69) and the performance of oral stereotypies which indicate the calves' environment is insufficient to meet their needs (70). Even still, it is standard practice to feed milk *via* open bucket or trough instead of a nipple or bottle throughout surplus production systems in the U.S. and Canada. Alternative feeding practices, such as providing milk through a bottle or an artificial nipple (71) may partially resolve these negative outcomes.

## Considerations for the Future of Surplus Dairy Animals

Currently, surplus dairy calves face several challenges in early life compromising their health, welfare, and the sustainability of the dairy and surplus calf industries. Here we offer both short and long-term recommendations for improving the lives of surplus dairy calves. In the short-term, we suggest dairy producers and calf raisers adopt practices that improve care of young animals, drawing from research using both dairy heifer calves and surplus calves. We also describe alternatives to the current system, such as direct to farm marketing and breeding for dairy-beef. In the long-term, we recommend the dairy industry develop a vision for the future of surplus animals that is sustainable.

## Short-Term Changes of the Current System

### Management From Arrival at Calf Rearing Facilities

Given health and welfare concerns, as well as concerning rates of antimicrobial use and resistance, the quality of calves arriving to calf raising facilities must improve. However, changes within calf raising facilities are also merited. Recent research has identified several calf characteristics measured at arrival to a calf-raising facility that were associated with an increased risk of morbidity and mortality as well as production losses. For example, clinical and blood measures could be used to identify and selectively manage calves that are deemed to be at high-risk for morbidity and mortality. Additional management changes at the veal or calf rearing facilities also need to be considered including providing a higher plane of nutrition, utilizing feed additives, and improving the housing environment.

### Using Clinical Parameters to Identify and Manage High-Risk Calves

Several clinical indicators assessed on arrival at a calf raising facility are associated with morbidity and/or mortality. Physical indicators include the presence of an abnormal fecal consistency, umbilical infection, dehydration, cough, and a sunken flank (2, 27). As these indicators are quick, simple, and have a reasonable repeatability following veterinary training, they could be used to create a selective therapy program in which calves arriving with the presence of these health abnormalities would be classified as high-risk and treated accordingly. A selective treatment strategy could lead to more prudent antimicrobial use and lead to a reduction in the use of blanket antimicrobials at arrival. This selective strategy was attempted by von Konigslow et al. (72), where a blanket oral antimicrobial strategy was compared to therapy provided to high-risk calves. No difference in morbidity was found during the first 14 days and there was a two-thirds reduction in antimicrobial use on arrival, however, calves in the selective therapy group had a greater risk of mortality compared to those that received blanket oral antimicrobials. This suggests that the use selective therapy requires further refinement to reduce antimicrobial use while still effectively reducing disease.

Body weight upon arrival has been consistently associated with future risk of morbidity and mortality, where calves with a higher body weight have a lower risk of disease (2, 50, 73). Arrival weight was also found to be the greatest influencer on the breakeven purchase price that should be paid for calves due to the lower risk of disease but also improved growth (74). Calf raisers should be encouraged to purchase calves that have a higher body weight; however, some portions of the current system inhibit the calf raiser from having complete control of purchasing calves with high body weight. Hence, until the issues from the source dairy farm are addressed (8, 10), calf raising facilities will need to develop strategies to manage calves arriving with a low body weight. Calves could be given a higher plane of nutrition, colostrum replacer or other bioactive compounds to improve gut health or potentially provided with antimicrobials. More work is needed to both increase the body weight of calves on arrival and ensure calves with low body weight are treated and monitored optimally.

### Using Blood Parameters to Identify and Manage High-Risk Calves

There has been a significant body of literature assessing the utility of blood parameters in predicting future disease risk in veal calves. Many parameters, such as haptoglobin, creatinine kinase, and cholesterol (29, 40, 73), are likely not realistic to make a rapid assessment of calves arriving on farm due to a lack of the availability of calf-side tests. There are, however, potentially practical calf-side tests that are available to identify calves at high-risk of disease based on serum proteins. For example, greater concentrations of immunoglobulin G (IgG) have been consistently associated with reduced disease occurrence in veal calves (29, 40, 75). On-farm IgG tests are becoming available which could allow for precise selection of calves with FTPI, however, the test performance has been variable when compared to radial immunodiffusion (3, 76, 77). Therefore, measuring serum total protein is likely the most accurate and accessible test available. The utility of this test to diagnosis individual calves with FTPI may be limited, however, it was found to correctly classify passive transfer status in 89% of calves at arrival to a veal facility (3). Managing calves with FTPI remains a challenge, but nutritional strategies, such as using colostrum supplementation, could be explored.

Recently, an on-farm machine leukocyte differential cell counter was validated (78) and used to predict disease, where calves with high levels of neutrophils or low levels of lymphocytes were at a greater risk of mortality (79). This on-farm machine could be used in combination to provide rapid risk assessment to identify and treat high-risk calves. Additional research is required in this area to determine the best strategy for managing these high-risk calves at either the individual or group-level.

### Nutritional Strategies

Creating customized nutritional strategies for calves with low body weight may be effective to mitigate disease risk and in general, increasing the plane of nutrition is a significant area for improvement. Calf raisers should focus on increasing the volume of milk provided to all calves to a minimum daily intake of 20% body weight in whole milk (80). Additionally, recent research suggests certain feed additives may aid in reducing the reliance on antimicrobials in calf-rearing systems. Specifically, supplementing colostrum and microbial-based probiotics and prebiotics has led to promising effects on health and growth. For example, supplementation with colostrum or colostrum replacer for the first 14 days of life has been shown to promote gastrointestinal health and weight gain while reducing antimicrobial use and disease prevalence in dairy heifers (81, 82). Even when supplemented for shorter durations, 2 to 4 days after birth, providing colostrum to dairy heifers improved weight gain and a decreased the risk of abnormal respiratory scores (83, 84). The benefits are likely related to antibacterial and antiviral lactoferrins and proinflammatory cytokines that can aid in combating infectious diseases in the gastrointestinal tract (85, 86). Supplementation with colostrum days after birth could be a promising option for calf raisers to reduce disease occurrence.

The use of microbial-based feed additives could also play a role in improving the gut health of young calves [reviewed by (87)]. Specifically, yeast supplementation during the pre-weaning period has been associated with a reduced incidence and severity of diarrhea in male dairy calves and calves raised for veal (88, 89), especially male calves with failed transfer of passive immunity (90). Supplementation with lactic acid bacteria has also been shown to reduce the risk of diarrhea [reviewed by (91)], particularly when male calves experienced high incidences of diarrhea (92, 93). There are, however, inconsistent results with supplementation (87), suggesting that other management practices may be important to consider. Nonetheless, the use of these microbial-based feed additives could be used in place of oral group antimicrobials provided to male and female calves as there is little evidence to support that practice (94).

### Improving Outcomes Associated With Transport

The best way to reduce negative outcomes from long-distance transportation is to eliminate transportation of young calves by raising animals on the dairy farm of birth until slaughter, or until calves are old enough to cope with transportation. In the event calves continue to be transported, the duration of transportation, number of stops, and exposure to severe weather conditions should be minimized. In addition to transportation conditions, some nutritional and therapeutic strategies may improve calf outcomes during and after transportation. For example, Marcato et al. (95) found calves fed milk (1.5 L) before 6 h transportation had greater plasma glucose and lower serum NEFA concentrations compared to calves given electrolytes (1.5 L); however, the authors found no treatment differences for calves transported for 18 h. Elevated NEFA and BHB concentrations are indicative of a negative energy balance, likely caused by feed deprivation. Feeding colostrum before transportation may reduce the depletion of body reserves compared to milk replacer because of the high fat and protein content (96). Depending on the length of transport and time spent at livestock auctions, it is not uncommon for calves to go without access to feed for more than 24 h. Ideally, young calves would not be transported longer than regular intervals between normal physiologic windows for nursing. However, limited research suggests that feeding a meal to calves immediately before transportation and at a regularly scheduled rest stop could reduce hunger and dehydration associated with transportation.

Another strategy that could potentially improve transportation outcomes is providing a non-steroidal anti-inflammatory drug before transportation. Non-steroidal anti-inflammatory drugs (NSAIDs) produce anti-inflammatory, anti-nociceptive and anti-pyretic effects. A study that assessed the administration of an NSAID (meloxicam) to young Jersey calves (≤ 3 d of age) before transportation, found calves that received meloxicam had greater feed intake and growth following arrival to a calf raiser, compared to calves that did not receive meloxicam (97). However, the sample size was small (*n* = 21) and calves were only monitored for 4 days after arrival to the production facility. Some of the health abnormalities observed on arrival, such as navel inflammation and elevated rectal temperature (27), may be prevented with an NSAID. Cost-effective and easily accessible therapeutic strategies could be quickly implemented as dairy farmers already have colostrum and milk replacer on farms, and frequently use NSAIDs on farm for calf-related purposes, such as dehorning.

### Benchmarking

One of the challenges directly related to surplus calf production is the lack of integration in the production chain. Many calves are marketed through auctions or third-party purchasers, leaving dairy producers with little to no knowledge of calf performance after removal from the dairy farm. However, providing dairy farmers with feedback regarding calf performance at calf raisers may motivate producers to improve animal care on the dairy. For example, Atkinson et al. (98) found dairy farmers made improvements to their colostrum management or milk feeding practices when they became aware of issues following the delivery of benchmark reports. Further, after providing dairy producers with benchmark reports of dairy heifer calf health and growth, producers identified challenges on their farm and made changes to directly address them (99). Similar to benchmarking, it may be beneficial for calves sold through auctions to have a record of their dairy farm of origin and/or details regarding early life care. Thus, we suggest increasing transparency between dairy farms of origin and calf purchasers and/or raisers may be a motivator to improve surplus calf care before removal from the dairy farm.

## Alternatives to the Current System

### Direct From Farm Purchasing

Auctions are a clear source of poor animal health and welfare for surplus calves (28). One alternative would be to avoid auctions and directly transport calves from the dairy farm of origin to a calf raiser. Eliminating marketing calves through livestock auctions could reduce the exposure of calves to environmental pathogens, decrease time spent being transported, and reduce stressors associated with auctions. Directly selling calves to buyers could also potentially improve calf care on the dairy farm. For example, Wilson et al. (18) found that dairy farmers in Ontario preferred selling calves directly to a purchaser instead of through an auction when possible and were motivated to maintain good relationships with direct calf buyers by supplying them with healthy calves. We encourage more work on possible barriers and opportunities for transporting calves directly from dairy farms to nearby calf raising facilities.

### Crossbreeding With Beef Animals

A second alternative to the current system is shifting toward breeding dairy cows with beef breeds to create cross-bred calves (e.g., Aberdeen Angus, Wagyu, and many others). There is currently little research on cross-bred calves, but the use of beef semen on dairy farms has reportedly grown substantially in recent years, such that beef breeds now represent 19 and 10% of semen used in dairy herds in 2019 in the U.S. and Canada, respectively (100, 101). In an exercise to envision global dairy farming in 2067, Britt et al. (102) anticipates breeding dairy cows differently depending on their genomic value; cows with high value will be bred using sexed semen for females to be raised as replacements, and cows with lower value will be bred to beef sires.

Researchers have considered dairy beef crossbreeding to have both economic and environmental benefits (103–105). For example, Pahmeyer and Britz (105) modeled the economic consequences of various breeding practices on German farms and found that breeding cows using sexed semen for replacement females and beef sires for surplus animals increased profits on average by €79.42 per cow per year. This increased profit is likely in part due to higher calf sale price based on the improved meat quality of cross-bred calves compared to purebred dairy beef animals (106). Holden and Butler (107) also describe the possible economic benefit of crossbreeding surplus animals, in addition to the potential reduction in greenhouse gases of this system compared to traditional beef. Researchers from New Zealand estimate a 29% decrease in greenhouse gases per kg in carcass weight from dairy-beef animals compared to traditional beef (108). As greenhouse gas emissions become regulated in various countries, the production of a lower impact dairy-beef animal may also be appealing to consumers attempting to reduce their carbon footprint.

The impact of crossbreeding on the health and welfare of calves is not well-understood. If crossbred calves are reared similarly to current purebred dairy calves used for beef and veal (e.g., shipped within a few weeks of age and co-mingled at new facilities or livestock auctions), the same concerns described in this review paper will still exist. However, there may be potential benefits to crossbreeding. For example, cross-bred calves may be considered a “higher value” animal due to their genetics. Increasing the monetary value of calves may in turn increase the motivation of the dairy farmers to take good care of these calves from birth.

### Raising Surplus Calves on Dairy Farms

Another potential refinement of the current system is to rear surplus calves on the dairy farm of origin, eliminating health and welfare challenges associated with long-distance transport and livestock auctions during the pre-weaning stage. In their vision of the dairy industry in 2067, Britt et al. (102) anticipates that future dairy farms will incorporate dairy-beef into existing or shared facilities. Rearing surplus animals on the farm of origin would require additional infrastructure and costs associated with rearing, but costs can be recovered by the sale of a high value animal later in life (105).

Retaining surplus animals on the dairy farm of origin would also allow dairy producers the option to transition to alternative dairy systems that allow for contact between the cow and her pre-weaned calf. Cow-calf contact systems are being studied as a form of housing that meets the growing public concern over the welfare of dairy animals (109). In two companion systematic review papers, Beaver et al. (110) and Meagher et al. (111) describe the concerns and advantages of this type of management system, including potential health challenges and improvements in affective states for cows and calves [e.g., improvement of emotional states in calves; (112)]. We encourage more research to identify options for cow-calf contact systems that incorporate surplus dairy animals.

### The Future of Surplus Dairy Animals

Like the rest of the dairy industry in the U.S. and Canada, decisions about the future of surplus dairy animals should be grounded in “sustainability” (113). Sustainability is a complex concept, but frameworks often include a balance of environmental, economic and social or ethical pillars. We argue that, for reasons described throughout this review paper, the current system for surplus dairy animals in the U.S. and Canada is not sustainable. A detailed assessment of the economic, environmental, and social impacts of various management systems for surplus dairy animals is outside the scope of this review. However, we recognize some “refinements” we recommend are not sustainable in the long term by these metrics.

If the current practices for surplus dairy animals remain unchanged, there are two main risks. First, policy changes beyond the control of the dairy industry may result in drastic management changes over a short period of time. For example, Canada recently introduced new transport regulations that will dramatically change the way some calves are moved. For many dairy producers, these new regulations will require calves to stay on the farm for longer periods than usual. There are currently no similar laws in the U.S. for pre-weaned calves, but it is possible that new regulations similar to those in Canada and the European Union will be implemented at some point by retailers. For example, some large-scale changes to farming practices in the United States swine and poultry industries have resulted from retailers responding to consumer and citizen concerns over animal welfare [e.g., elimination of gestation stalls and conventional cages; (114)]. The dairy industry is not immune to similar changes if management practices continue to be misaligned with public attitudes and values.

Secondly, the dairy and surplus calf industries are at risk of losing their “social license” to farm without government oversight. Social license refers to “the process by which a community grants or withholds permission to an industry to conduct its business” (115). That is, farmers are generally afforded the ability to make their own decisions about how to rear their animals. However, many current practices on dairy farms are “misaligned” with public values, resulting in distrust of the industry (116). Surprisingly little research has assessed public values about surplus dairy calves. When asked about food animal agriculture in general, the public strongly values naturalness, such as pasture access as well as indoor environments that allow for the expression of natural behavior, freedom of movement and socializing with companions [reviewed by (117, 118)]. The current system for rearing surplus dairy calves is heavily reliant on unnatural housing (e.g., indoor housing with mechanical ventilation or outdoor housing with low space allowance), isolated social environments (e.g., individual pens), and inadequate feeding programs (low milk allowances compared to what they would drink from the dam) which are in direct contrast to public values.

A main aspect of current surplus calf management that is misaligned with public views is the practice of euthanizing healthy newborn calves. In the UK, several organizations are opting to ban the routine euthanasia of surplus dairy calves, likely in response to public concerns about the practice (119). This response is not surprising, given that the public has responded similarly to the euthanasia of healthy surplus zoo animals [e.g., Marius the giraffe; (120)] as well as male chicks in the egg laying industry (121). Ethical concerns over the mass culling of healthy male chicks has resulted in a ban of this practice in France, Switzerland, and Germany, leaving the egg laying industry to find alternative management solutions. Thus, if the dairy industry in the U.S. and Canada does not proactively find alternatives to the routine euthanasia of surplus dairy calves, changes to this practice may occur top-down.

To avoid these risks, we recommend the dairy and associated industries in the U.S. and Canada take a pro-active approach to the fate of surplus dairy calves. This approach should consider viewpoints from multiple stakeholders both within and outside of the dairy industry. For example, Weary and von Keyserlingk (116) recommend engaging with the public over controversial issues within the dairy industry using qualitative social science research. Understanding public expectations can help inform decision-making that promotes sustainable practices. Other social science and mixed methods approaches that have been used to help resolve complex issues are also recommended, such as deliberative democracies (122), participatory research including dairy farmers (123) and sustainability science (124). A qualitative research approach is also needed to understand the motivations and barriers to adoption of best management practices for dairy, veal, and other calf raisers (18, 99). Determining how to encourage producers to adopt new management practices for surplus calves will likely be key to seeing industry wide changes. Ideally, a diverse research approach can help the dairy industry construct a vision for surplus animals that meets the needs of multiple stakeholders centered around improving calf health and welfare.

## Conclusion

Approximately half of calves born to dairy cows, including all male and non-replacement female calves, are sold from the dairy farm to calf-raisers within the first few days to weeks of life. Sub-optimal care of surplus calves generally begins at birth and continues throughout production. Surplus calf management practices during early life include poor colostrum management, long-distance transportation, marketing through livestock auctions, individual housing, and low planes of nutrition. Poor treatment of calves likely results in negative affective states, and high rates of morbidity and mortality. Short-term changes to surplus calf production including minimizing transportation and eliminating marketing calves through livestock auctions, crossbreeding, and raising calves on the dairy farm are options to improve calf outcomes. In the long-term, a holistic approach that takes producer perspectives, social concerns, industry viewpoints, and calf outcomes into account is needed to redesign a sustainable future for surplus calves.

## Author Contributions

KC, JP, GH, SL, KP, DW, and DR: conceptualization. KC, JP, GH, SL, KP, and DR: investigation and writing—original draft. GH and DR: funding acquisition and supervision. All authors contributed to the article and approved the submitted version.

## Conflict of Interest

The authors declare that the research was conducted in the absence of any commercial or financial relationships that could be construed as a potential conflict of interest.
